# ﻿*Thismialimkokthayi* (Thismiaceae): A new mycoheterotrophic species from Genting Highlands in Pahang, Malaysia

**DOI:** 10.3897/phytokeys.211.89453

**Published:** 2022-10-11

**Authors:** Mat Yunoh Siti-Munirah, Mustafa Suhaida, Chan Eddie

**Affiliations:** 1 Forest Research Institute Malaysia, 52109 Kepong, Selangor, Malaysia Forest Research Institute Malaysia Kepong Malaysia; 2 Awana Hotel Genting Highlands Resort, 69000 Genting Highlands, Pahang, Malaysia Awana Hotel Genting Highlands Resort Genting Highlands Malaysia

**Keywords:** fairy lantern, Genting Highlands, taxonomy, upper hill dipterocarp forest

## Abstract

*Thismialimkokthayi*, a distinct mitriform species of the mycoheterotrophic genus *Thismia*, is described and illustrated. It was found at a locality in the upland areas of Genting Highlands, Pahang, Malaysia. This new species is morphologically similar to members of Thismiasect.Geomitra, but differs in several characteristics, including the colour of the floral tube, the inner surface of the floral tube with longitudinal ribs and absent transverse bars, a stamen apex with a central lobe (prolongation of the rib) and two lateral lobes (the tips of each are recurved) and a black-purplish stigma. *Thismialimkokthayi* is provisionally classified as Critically Endangered according to the IUCN Red List Categories and Criteria.

## ﻿Introduction

Fairy lanterns, *Thismia* Griff. (1844) (Thismiaceae), are non-photosynthetic mycoheterotrophic herbs distributed mainly in the tropics. The number of known species of this genus has increased rapidly in recent years. Currently, approximately 96 species are known (Imhof 2010 onwards; Siti-Munirah et al. 2021; Chantanaorrapint and Seelanan 2021; Siti-Munirah and Dome 2022). To date, three species of *Thismia* are known in Pahang State: *T.alba* Holttum & Jonker, *T.aseroe* Becc. and *T.racemosa* Ridl. (Jonker 1948; Chua and Saw 2006). Morphologically, all of these species belong to Thismiasect.Thismiasubsect.Odoardoa, in which all the tepals are fully open, spreading and not forming any mitre as in sect. Geomitra.

During a routine assessment of work progress near the Clearwater Way trail, located in a private forest not far from the Resorts World Genting Awana Hotel (Genting Highlands, Bentong District, Pahang), an unknown plant was discovered. The discovery was made on 1 April 2022, by the third author, who observed the unknown plant in a population growing on the nature trail. Based on images sent to the first author for identification, it was suspected to be a new taxon of *Thismia*. Later (7 April 2022), we visited the site together and were able to find additional plants within the same population. Several plants were collected for the herbarium and for taxonomic study. After careful examination, the specimens were found to have some novel features in terms of flower tube and tepal morphology. These features formed a unique combination of characteristics that were not matched with any of the described species of *Thismia*. Therefore, we now describe a new species that we have named *Thismialimkokthayi* Siti-Munirah & E.Chan. This additional new species brings the number of currently known species of *Thismia* in Pahang to four. This discovery has also resulted in *T.limkokthayi* becoming the first mitriform (*Geomitra*) species reported in the State of Pahang.

## ﻿Materials and methods

**Map 1.**
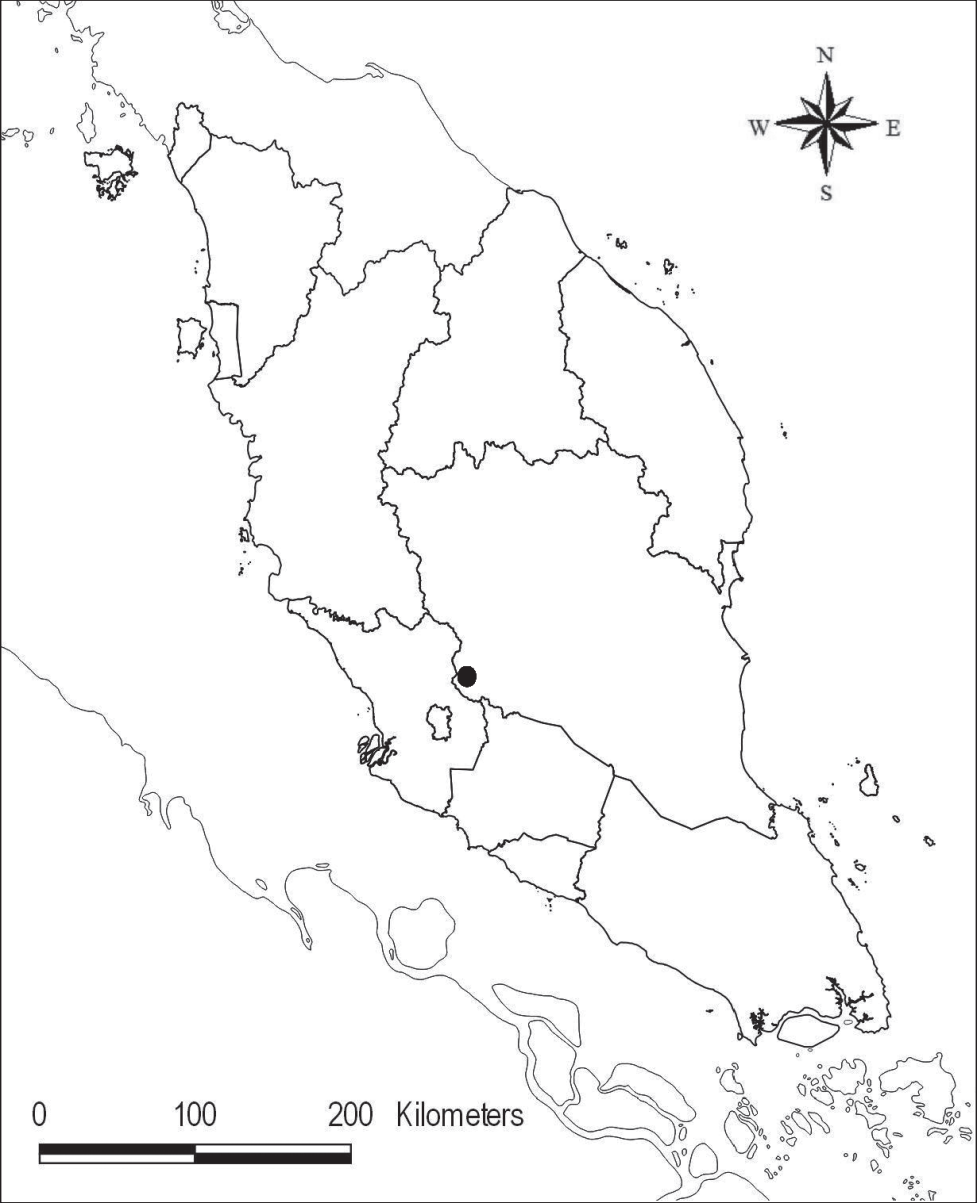
Map of the Malay Peninsula indicating the location of the Genting Highlands (dot), the type locality *of Thismialimkokthayi*.

This study is based on material collected on 7 April 2022 from Genting Highlands Forest, Bentong District, Pahang (Map 1), on private land near the Resorts World Genting Awana Hotel, which is one of the flagships for tourism in the mountains of this region. Genting Highlands is a famous mountainous region in Bentong District, Pahang, located approximately 50 km from the capital of Malaysia, Kuala Lumpur. Genting Highlands is renowned for entertainment, hospitality and tourism, as well as nature. As an area with an exceptional diversity of flora and fauna, it is home to a significant reservoir of primary rainforest (more than 15,000 acres); however, part of the area is fully developed.

One of the collected individuals was observed during its flowering growth (in the office at the Forest Research Institute Malaysia (FRIM)) and was selected as holotype (FRI91138c) due to its perfect condition. Morphological characteristics were examined by stereomicroscopy and high-resolution macrophotography. Measurements were made on fresh and liquid preserved material. The specimen was thoroughly compared with original drawings and descriptions in the protologues of Thismiasect.Geomitra.

## ﻿Taxonomic account

### ﻿ Thismia limkokthayi

Taxon classificationPlantaeDioscorealesThismiaceae

Siti-Munirah & E.Chan sp. nov.

0C02AA5A-E1A1-500D-A649-5FD2F2626DD2

urn:lsid:ipni.org:names:77306479-1

Figs 1 , 2 , 3

#### Diagnosis.

*Thismialimkokthayi* is distinguished from closely-related species by the following combination of characteristics: black to dark brown floral tube and appendages, the presence of mitre fovea between the bases of claviform appendages, the presence of longitudinal ribs and absent transverse bars on the inner side of the floral tube, the apex of stamens with a central lobe (prolongation of the rib) and two lateral lobes (the tips of each are recurved) and dark purple stigma.

#### Type.

Malaysia. Peninsular Malaysia: Pahang, Bentong District, Genting Highlands, ca. 1137 m elev., 28 April 2022, Siti-Munirah MY, FRI91138c (holotype KEP!, spirit collection, barcode no. SC12022).

Achlorophyllous herbs up to ca. 14 cm. **Roots** coralliform, surface hairy, apices brownish-white. **Stems** up to ca. 4.5 cm tall or very short (possibly for young plants), ca. 2–3 mm in diameter, erect, ascending, white and becoming brownish with age, glabrous, terete. **Leaves** glabrous, pale brown, dark brown in the upper part or towards the apex, scale-like, triangular-ovate to lanceolate, up to 12 mm long, 3 mm wide at the base, apex acute to acuminate, spirally arranged, more crowded in the upper part of the stem. **Floral bracts** 3, pale brown to dark brown towards the apex, similar to upper leaves, but slightly larger, 10–12 mm long, apex acute to acuminate, 2 mm wide at the base. **Pedicels** lengthen up to 5 mm during flower growth, 2–3 mm wide. **Flowers** solitary or in clusters of 2, opening in succession in the latter case and forming loose monochasial inflorescences, lateral flower located in the axil of one of the floral bracts of the terminal flower and also bearing its own floral bracts; each flower up to 8 cm long (including ovary and appendages); **perianth** actinomorphic with 6 tepals fused to form a floral tube with a dome-shaped mitre with 3 slender, claviform appendages on its top; **floral tube** black brownish/brown blackish, urceolate, ca. 20–25 mm long, ca. 5–10 mm wide, constricted just above the ovary, widest in the upper part; **outer surface** with 6 longitudinal ribs, glabrous, rough, black to dark brown; **inner surface** with 6 greenish longitudinal ribs, without transverse bars, black to dark brown; **outer tepal lobes** 3, brownish orange, minute, ca. 1 mm long, 7 mm wide at base, broadly triangular, erect; **inner tepal lobes** 3, black to dark brown, thick, cuneate, surface glabrous, apically adnate to form a dome-shaped mitre; **mitre** with 3 lateral, round-shaped, ca. 8–10 mm wide apertures, 3 hood-like accessory lobes, more curved during the early stages of flowering and flattening when the flower is older and matured; **mitre appendages** each ca. 27–30 mm long, their base wide and flattened, forming a fovea in the centre of the mitre, becoming narrower above, claviform at apex, glabrous, dark brown to pale orange towards tip. **Stamens** 6, pendulous from the apical margin of the floral tube; **annulus** absent; **filaments** orange and white, curved downwards, with bases slightly emerged above floral tube apex, not connate, forming 6 apertures apparent when viewed from above; **connectives** broad, orange-yellowish in lower half and black/dark brown in upper half, laterally connate to form a tube, ca. 12 mm long, each with prominent longitudinal rib extending along the entire length of the inner surface of the connective; supraconnective apex with one central lobe (extension of the rib) and two smaller side lobes with tips recurved inwards/truncate, glabrous; **lateral appendage** skirt-like, black, protruding towards the floral tube, not reaching connective apex, glabrous including on margins, only sparsely hairy on each horn-like corner; individual **stamens** with 2 thecae (abaxial, dehiscing towards the inner surface of the floral tube), each **theca** oblong, 2 mm long; **interstaminal glands** elliptic-oblong, translucent, inserted on the line of fusion between connectives. **Ovary** inferior, obconical, ca. 4–5 mm long, brown to dark, with 6 longitudinal ribs, unilocular; **placentas** 3; **style** short, ca. 0.3 mm long, black/purplish; **stigma** 3-lobed, black-purplish, **stigma lobe** oblong, ca. 3 mm long, folded, bifid at apex, surface slightly papillose. **Fruit** not observed.

#### Additional specimens examined (paratypes).

Peninsular Malaysia: Pahang, Bentong, Genting Highlands, ca. 1137 m elev., 7 April 2022, Siti-Munirah MY, Eddie C & Suhaida M, FRI91138a (KEP, spirit collection, barcode no. SC12021); 7 April 2022, Siti-Munirah MY, Eddie C & Suhaida M, FRI91138b (KEP, spirit collection, barcode no. SC12020)

#### Distribution.

Endemic to Peninsular Malaysia, Pahang. Currently known only from the type locality.

**Figure 1.**
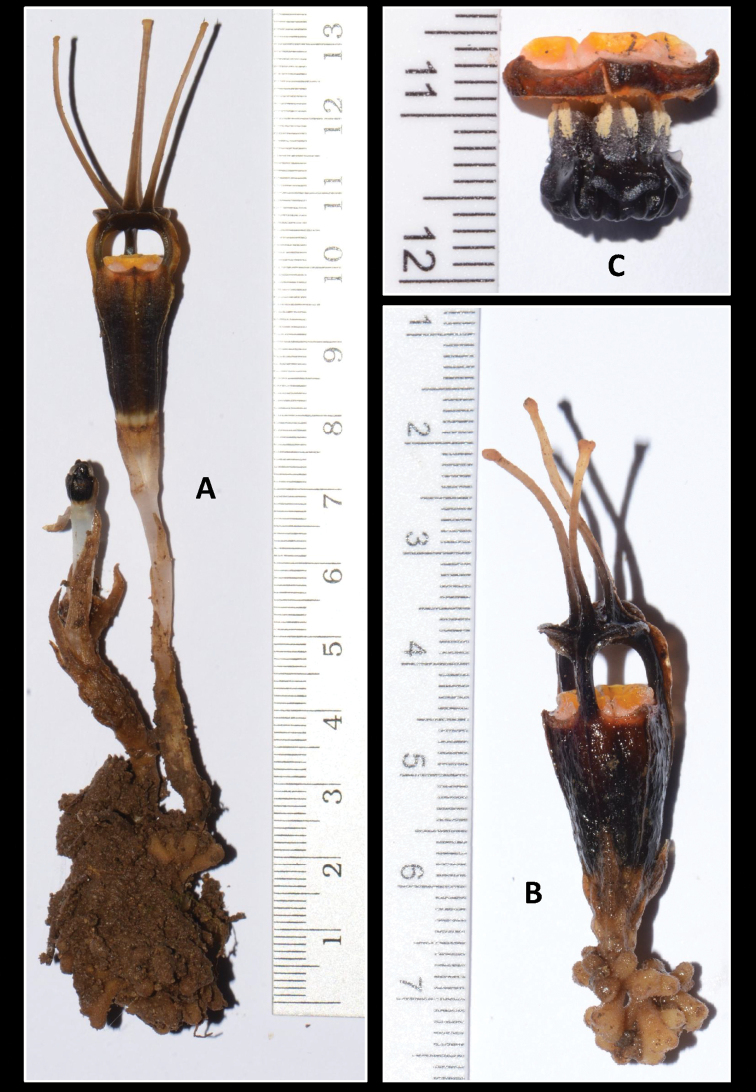
*Thismialimkokthayi* with scale (all in centimetres (cm)) **A** plants with mature (FRI91138c) and young flowers with long stems **B** mature flower with a very short stem (FRI91138b) **C** stamens (FRI FRI91138a). Photos by Siti-Munirah MY.

**Figure 2.**
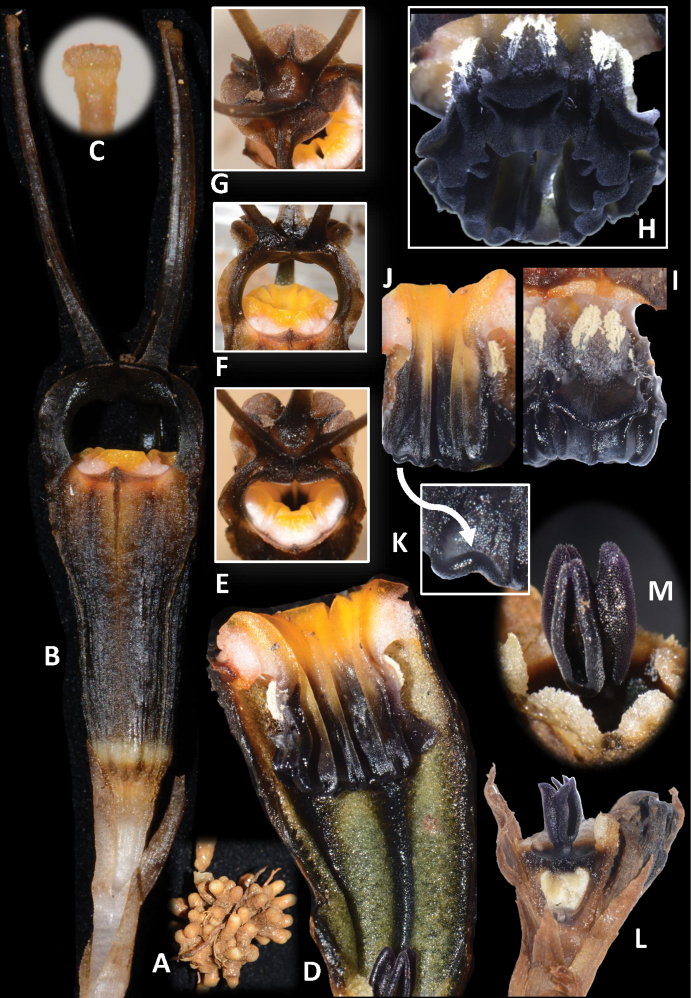
*Thismialimkokthayi***A** coralliform roots **B** full flower **C** claviform tip of mitre appendage **D** longitudinal section of floral tube, showing inner surface **E** outer tepal and inner tepal **F** side view of mitre **G** mitre viewed from above **H** six stamens, viewed from below **I** outer view of a stamen **J** inner view of two pendulous stamens **K** detail of the side lobe on the supraconnective apex (side lobe with truncate tips that are recurved inwards) **L** ovary with style, stigma and flower bud surrounded by bracts **M** stigmas. All from FRI FRI91138a (**A, D, I, J, K, L, M**), FRI91138b (**H**) and FRI91138c (**B, C, E, F, G**). Photos by Siti-Munirah MY; images not to scale (see dimensions in description and Fig. 1).

#### Ecology and habitat.

**Figure 3.**
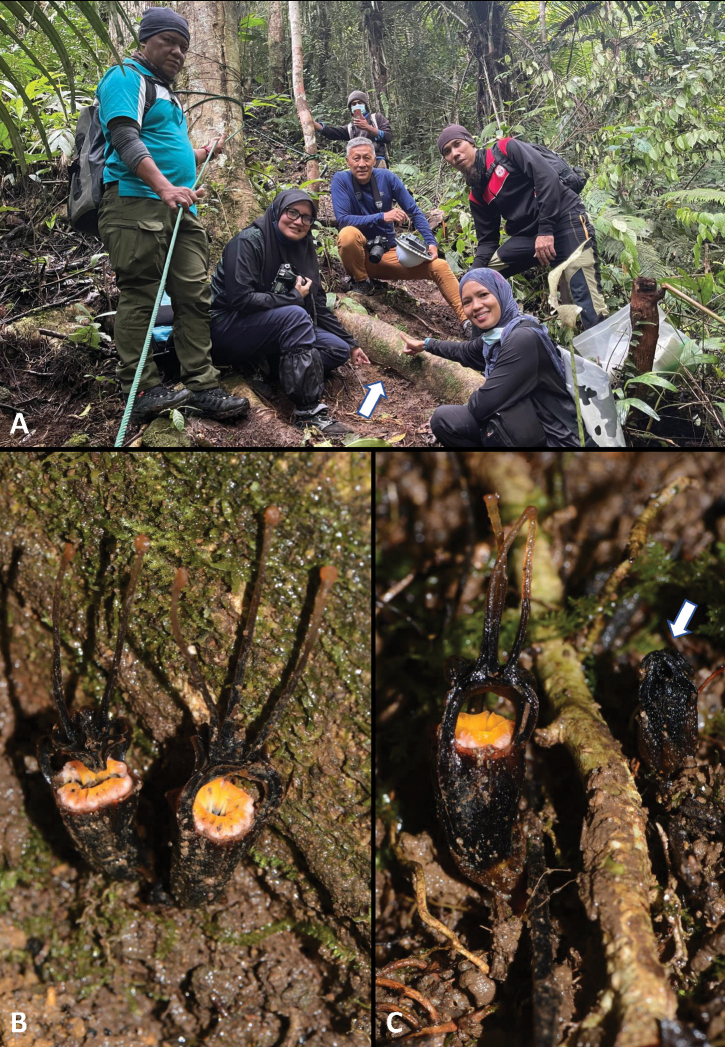
*Thismialimkokthayi* Siti-Munirah & Eddie Chan **A** group photo with the *T.limkokthayi* (white arrow) in its habitat (from left; Angan A, Siti-Munirah MY, Eddie C, Ahmad Norsidar AH and Suhaida M) **B–C** different stages of anthesis of *T.limkokthayi* in its habitat (in situ) **B** two mature flowers from one individual **C** a fresh flower (on the left) and a new young flower starting to bloom (on the right). Photos by Siti-Munirah MY.

In lower montane forest and upper dipterocarp forest, on moist soil in shade, near an open space (hiking trail; Fig. 3A) and sloping area. This species occurs in a healthy undisturbed forest at an altitude of about 1137 m. The site is in a private forest and within a watershed. The forest area remains intact, apart from where it was affected by recent flooding and water surge (19 December 2021). The impact of this natural disaster completely reshaped part of the river area. However, this area is currently recovering. Fortunately, the only known population of *Thismialimkokthayi* is located away from the riverbank, on the slope of the main trail. As a result of this discovery, the trail was moved to another part of the forest. The flowering period is from March to April.

#### Etymology.

*Thismialimkokthayi* was named in honour of Tan Sri Lim Kok Thay, Chairman of the Genting Group, who is closely involved in efforts to develop eco-tourism facilities and amenities supporting the preservation and sustainability of important biodiversity assets and sites in Genting Highlands.

#### Preliminary conservation status.

Following the IUCN Red List Categories and Criteria (IUCN 2019), this species has been assessed as Critically Endangered (CR B2ab (iii); D) due to its small population and threats to its microhabitat. It is currently known from only one locality (the type locality) and is certainly a very rare species since only three individuals have been observed. Although the type locality is in a private forest, it may be exposed to future tourist activities in the Resorts World Genting Awana area. The habitat of the species is located on the original trail leading from the entrance of Clearwater Way to Chin Swee Caves Temple. However, since this is private land, the protection of this area from disturbance remains possible. As such, efforts must be made to locate this species in the surrounding area.

#### Notes.

*Thismia* species are characterised by their peculiar appearance and flower morphology, which are distinctive and simultaneously very diverse (Shepeleva et al. 2020). Most *Thismia* species have various unique structural combinations. Amongst them, there are species belonging to the *Thismia* group of which inner tepals are fused together in their upper part and form a roof-like or hat-like structure called a mitre. In Peninsular Malaysia, there are two groups of mitriform *Thismia*: first including *Thismia* species with mitre appendages (e.g. *Thismiaclavigera*, *Thismiakelantanensis* and *T.clavigeroides*) and second including *Thismia* species without appendages (e.g. *T.latiffiana* Siti-Munirah & Dome and *T.sitimeriamiae* Siti-Munirah, Dome & Thorogood).

*Thismialimkokthayi* is easily recognised by a combination of the following characteristics: coralliform roots, erect outer perianth lobes, mitriform inner perianth lobes with three erect, slender, claviform appendages that form a mitre fovea with their flattened bases in the centre, absence of the annulus, presence of longitudinal ribs on the inner side of the floral tube (or absence of the reticulate inner side), the apex of stamens with a central lobe (prolongation of the rib) and two lateral lobes (the tips of each are recurved) and black-purple stigma.

Based on the infrageneric classification of Kumar et al. (2017), *T.limkokthayi* superficially resembles *Thismia* species in the ThismiasubgenusThismiasectionGeomitra (Becc.) Kumar & S.W. Gale, mainly based on the presence of inner tepals forming a mitre, each with filiform appendage and central mitral appendages that are free from each other, with the outer tepals always being short (less than 2 mm long). Two species were included in section Geomitra: *T.clavigera* (Becc.) F. Muell. and *T.betung-kerihunensis* Tsukaya & H. Okada.

To date, *T.limkokthayi* has not been included in any DNA-based analysis. According to the classification proposed by Shepeleva et al. (2020), *T.limkokthayi* is possibly related to species in clade 3 because it is characterised by coralliform roots, inner tepals fused into a mitre and free mitre appendages extending from a central point. Within clade 3 (i.e. section Sarcosiphon sensu Jonker 1948), there are two morphologically distinct groups characterised by the presence of a prominent central rib along the inner side of the connective and the absence of an annulus (Dančák et al. 2020). They can be distinguished by the presence of distinct appendages at the top of the mitre (*T.clavigera* group) or their absence (*T.goodii* group). However, their exact relationship cannot be clarified until more species in both groups are sequenced.

Currently, there are five species known in the *T.clavigera* group: *T.betung-kerihunensis*, *T.clavigera*, *T.clavigeroides* Chantanaorr & Seelanan, *T.kelantanensis* Siti-Munirah and *T.sumatrana* Suetsugu & Tsukaya (Tsukaya and Okada 2012; Siti-Munirah 2018; Suetsugu et al. 2018; Chantanaorrapint and Seelanan 2021). *Thismialimkokthayi* clearly differs from all these species by the absence of transverse bars on the inner surface of the floral tube. Notably, *T.limkokthayi* clearly resembles *T.kelantanensis*, an endemic species from Kelantan (Malay Peninsula), which also has three slender, claviform appendages at the tip of the mitre and erect outer tepals. However, *T.kelantanensis* is easily distinguished from *T.limkokthayi* by its bluish filaments and stamens, as well as the six-part cap on the mitre. Additionally, it also resembles *T.clavigeroides* Chantanaorr & Seelanan from Thailand and *T.sumatrana* Suetsugu & Tsukaya from Sumatra in its general appearance. However, *T.limkokthayi* differs from both of these species by the curved outer lobes on the supraconnective apex. Moreover, the connective appendage of *T.sumatrana* is hairy, while it is glabrous in *T.limkokthayi* and the flower colour of *T.clavigeroides* (including the stamens) is almost whitish, while that of *T.limkokthayi* is blackish-brown. A comparison of morphological characteristics between *T.limkokthayi* and other related species is presented in Table 1.

**Table 1.** Morphological differences between *Thismialimkokthayi* and related species. The characters of previously-described species are taken from the protologues and recent publications on *T.betung-kerihunensis* (Tsukaya and Okada 2012), *T.clavigera* (Chantanaorrapint and Chantanaorrapint 2009), *T.kelantanensis* (Siti-Munirah 2018) and *T.sumatrana* (Suetsugu et al. 2018).

Characters	*T.limkokthayi*	*T.betung-kerihunensis*	*T.clavigera*	*T.clavigeroides*	*T.kelantanensis*	*T.sumatrana*
**Colour of floral tube**	Black-brownish/brown-blackish	White with indigo and brown to pale brown with purple-dark blue	White-orangish	Whitish	Pale to bright and dark blue-purplish translucent,	Unknown
**Colour of mitre**	Black-brownish/brown-blackish	Blue-green	Yellowish-orange	Pale brown or grey	Yellow to bright orange	Unknown
**Colour of inner tepal lobes**	Brown-blackish	Blue-green	Orange	Pale brown or grey	Bright orange	Unknown
**Outer tepal**	Erect	Erect	Erect	Reflexed	Erect	Reflexed
**Colour of filament**	Orange and white	Blue-green	Orange	White	Bright blue	Unknown
**Colour of appendages**	Dark brown to pale orange	Pale blue tinged with orange	Orange	Pale brown or grey below, blue-green at tip	Orange	Unknown
**Fovea**	Present	Absent	Absent	Absent	Absent	Absent
**Apex of supraconnective**	One central lobe (extension of the rib) and two smaller side lobes with tips recurved inwards/truncate, glabrous	One central lobe (extension of the rib) and two smaller side lobes, hairy	Acute	One central lobe (extension of the rib) and two smaller side lobes	Acuminate	Acute, hairy
**Presence of transverse bars**	Absent	Present	Present	Present	Present	Present

## Supplementary Material

XML Treatment for Thismia limkokthayi
